# Retinal perivascular amyloid imaging in relationship to MRI neuroimaging abnormalities and cognitive impairment

**DOI:** 10.1002/alz.090891

**Published:** 2025-01-09

**Authors:** Oana M Dumitrascu, Jonah Doustar, Dieu‐Trang Fuchs, Yosef Koronyo, Dale S. Sherman, Michelle Miller, Ken Johnson, Roxana O. Carare, Steven R Verdooner, Patrick Lyden, Julie A. Schneider, Keith L Black, Maya Koronyo‐Hamaoui

**Affiliations:** ^1^ Mayo Clinic College of Medicine and Science, Scottsdale, AZ USA; ^2^ Department of Neurosurgery, Maxine Dunitz Neurosurgical Research Institute, Cedars‐Sinai Medical Center, Los Angeles, CA USA; ^3^ Cedar SInai Medical center, Los Angeles, CA USA; ^4^ Cedars Sinai Medical Center, Los Angeles, CA USA; ^5^ Cedars‐Sinai Medical Center, Los Angeles, CA USA; ^6^ Tulane University School of Medicine, New Orleans, LA USA; ^7^ Neurovision Inc, Sacramento, CA USA; ^8^ University of Southampton, Southampton UK; ^9^ NeuroVision Imaging, Inc, Sacramento, CA USA; ^10^ University of Southern California, Los Angeles, CA USA; ^11^ Department of Neurology and Pathology, Rush University Medical Center, Chicago, IL USA; ^12^ Department of Neurology, Cedars‐Sinai Medical Center, Los Angeles, CA USA; ^13^ Department of Biomedical Sciences, Division of Applied Cell Biology and Physiology, Cedars‐Sinai Medical Center, Los Angeles, CA USA

## Abstract

**Background:**

Scanning laser ophthalmoscopy (SLO) visualizes two important markers of cognitive dysfunction in the retina: vascular changes and amyloid plaque (AP) deposition. The relationship between retinal arteriolar versus venular changes and perivascular amyloid deposition across the continuum of neurodegeneration is imperfectly understood. We investigate the retinal perivascular AP distribution in relationship to cognitive and neuroimaging measures in a cohort of cognitively normal and impaired individuals.

**Method:**

34 subjects with normal or impaired cognition underwent retinal SLO, brain magnetic resonance imaging and standard neuropsychometric testing. Retinal peri‐arteriolar and peri‐venular curcumin positive AP were quantified in the supero‐temporal retinal quadrant, in the first, second and third‐order retinal vascular branches. The peri‐venular and peri‐arteriolar AP count were compared between patients with normal and impaired cognition, and their correlation with brain volumes, white matter hyperintensities and cognitive Z‐scores was determined.

**Result:**

We analyzed retinal para‐venous and para‐arteriolar amyloid count in 28 subjects, mean (SD) age 65 (7.3), 50% female, 19 (52.6%) with impaired cognition. Para‐arteriolar plaque count was higher than peri‐venular plaque in the entire cohort (p<0.0001). Secondary branch peri‐venular amyloid plaque count was higher in patients with MOCA ≤26. Tertiary branch perivascular AP count significantly and negatively correlated with hippocampal volume and positively correlated white matter hyperintensity lesion count.

**Conclusion:**

Retinal AP deposit peri‐arteriolarly overall and peri‐venularly in the secondary branches in cognitively impaired. Perivascular amyloid burden in small retinal branches correlates with hippocampal volume and white matter hyperintensities.

